# Bilateral kidney preservation by volumetric-modulated arc therapy (RapidArc) compared to conventional radiation therapy (3D-CRT) in pancreatic and bile duct malignancies

**DOI:** 10.1186/1748-717X-6-147

**Published:** 2011-10-31

**Authors:** Sabine Vieillot, David Azria, Olivier Riou, Carmen Llacer Moscardo, Jean-Bernard Dubois, Norbert Aillères, Pascal Fenoglietto

**Affiliations:** 1 Département de Cancérologie Radiothérapie et de Radiophysique, CRLC Val d'Aurelle-Paul Lamarque, Montpellier, France

**Keywords:** volumetric-modulated arc therapy, rapidarc, pancreatic cancer

## Abstract

**Background:**

To compare volumetric-modulated arc therapy plans with conventional radiation therapy (3D-CRT) plans in pancreatic and bile duct cancers, especially for bilateral kidney preservation.

**Methods:**

A dosimetric analysis was performed in 21 patients who had undergone radiotherapy for pancreatic or bile duct carcinoma at our institution. We compared 4-field 3D-CRT and 2 arcs RapidArc (RA) plans. The treatment plan was designed to deliver a dose of 50.4 Gy to the planning target volume (PTV) based on the gross disease in a 1.8 Gy daily fraction, 5 days a week. Planning objectives were 95% of the PTV receiving 95% of the prescribed dose and no more than 2% of the PTV receiving more than 107%. Dose-volume histograms (DVH) for the target volume and the organs at risk (right and left kidneys, bowel tract, liver and healthy tissue) were compared. Monitor units and delivery treatment time were also reported.

**Results:**

All plans achieved objectives, with 95% of the PTV receiving ≥ 95% of the dose (D95% for 3D-CRT = 48.9 Gy and for RA = 48.6 Gy). RapidArc was shown to be superior to 3D-CRT in terms of organ at risk sparing except for contralateral kidney: for bowel tract, the mean dose was reduced by RA compared to 3D-CRT (16.7 vs 20.8 Gy, p = 0.0001). Similar result was observed for homolateral kidney (mean dose of 4.7 Gy for RA vs 12.6 Gy for 3D-CRT, p < 0.0001), but 3D-CRT significantly reduced controlateral kidney dose with a mean dose of 1.8 Gy vs 3.9 Gy, p < 0.0007. Compared to 3D-CRT, mean MUs for each fraction was significantly increased with RapidArc: 207 vs 589, (p < 0.0001) but the treatment time was not significantly different (2 and 2.66 minutes, p = ns).

**Conclusion:**

RapidArc allows significant dose reduction, in particular for homolateral kidney and bowel, while maintaining target coverage. This would have a promising impact on reducing toxicities.

## Background

Over the last decades, progress in treating pancreatic cancer has remained modest, and the disease is still associated with very poor prognosis regardless of stage. Although there is currently no clear standard therapy, chemotherapy (CT) alone or radiochemotherapy (RCT) are two treatment options available for patients with non metastatic locally advanced pancreatic cancer. Two systematic reviews have shown no difference between both treatment modalities in terms of overall survival, but an increased toxicity with RCT [1,2]. Lack of benefit for RCT can be explained by the low compliance related to acute toxicity. In a recently published phase 3 trial comparing intensive induction chemoradiation followed by gemcitabine to gemcitabine alone, Barhoumi et al. reported that only 42% of patients were able to receive 75% of the planned chemoradiation schedule (60 Gy, 2 Gy/fraction and concomitant infusion of 5-fluorouracil and cisplatin) whereas 73% of patients were given 75% of the treatment of gemcitabine alone (1,000 mg/m2 weekly) [3]. Interestingly, the administration of induction chemotherapy before RCT has been shown to be a promising strategy for selected patients with non progressive disease, which may help to define the subset of patients likely to benefit from RCT while sparing those with rapidly progressive disease from potentially toxic radiotherapy [4]. In the case of resectable pancreatic cancer, it is now well established that RCT should be only considered in the adjuvant setting after insufficient pancreatic resection [5], whereas for bile duct malignancies, radiotherapy (RT) is likely to prolong survival in case of locally advanced disease as well as after R1 resection [6].

The occurrence of toxicity is clearly associated with the use of old RT techniques and the delineation of important target volumes. Besides, recent data regarding the treatment of pancreatic disease are in favor of RCT without prophylactic irradiation of peripancreatic lymph nodes since the use of small radiation fields seems to provide similar local recurrence rates and lower gastrointestinal (GI) toxicity [7,8]. The goal of modern radiotherapy such as the intensity modulated radiation therapy (IMRT) or the volumetric-modulated arc therapy (VMAT) is to insure full delivery of the prescribed dose, even to allow dose escalation while reducing doses to surrounding critical organs (small bowel, kidneys, spinal cord). A few studies investigated IMRT for pancreatic cancers and showed a statistically significant improvement over three dimensional conformal radiotherapy (3D-CRT) in lowering dose to the liver, stomach and bowel [9-12]. Therefore, advances in RT delivery with IMRT suggest a tolerability advantage including a lower incidence of acute toxicity [13,14]. In addition to a favorable toxicity profile, the local control is not compromised with IMRT [15].

Volumetric-modulated arc therapy (RapidArc) is a new form of IMRT optimization combining one gantry rotation and the capability to vary dose-rate, gantry speed and dynamic multi leaf collimator (MLC) [16]. Details of the RapidArc process and specific quality assurance procedures have been described in several publications [16,17]. The VMAT approach has a number of potential advantages compared to IMRT by significantly reducing the treatment time and the number of monitor units (MU), and improving normal tissue sparing while keeping the adequate target coverage [18-20]. In a trial comparing VMAT and IMRT for pancreatic cancers, VMAT was shown to provide superior conformity indices to the target and to reduce the dose in organs at risk (OAR) volumes with a shorter delivery time [21].

The purpose of the present study was to perform a comparative dosimetric analysis of RapidArc and 3D-CRT techniques in the treatment of pancreatic and bile duct malignancies with a special focus on the preservation of bowel and bilateral kidney function.

## Methods and materials

### Treatment plans

Treatment plans from 21 consecutive patients previously treated in our institution for pancreatic cancer (n = 17) or cholangiocarcinoma (n = 4) were used for this study. Among patients with pancreatic cancer, 14 patients had undergone radiation therapy for unresectable locally advanced disease, 2 for local recurrence after curative resection and 1 for adjuvant treatment due to insufficient surgical resection. Cholangiocarcinoma treatment plans were obtained from 2 patients with locally advanced disease, one patient with local recurrence and one having received adjuvant therapy.

The gross tumor volume (GTV) was determined by CT-scan and/or position emission tomography (PET) scan, and was defined as the tumor and enlarged regional lymph nodes. Clinical target volume (CTV) consisted of microscopic extensions around the GTV, as well as the anastomotic site and surgical clips in case of adjuvant treatment. When a 4D-CT scan was used, an internal target volume (ITV) was created to account for respiratory motion in 3 dimensions. The planning target volume (PTV) was a 0.5-1 cm expansion of the CTV (and internal target volume (ITV) if existing). The OAR included bowel, spinal cord, liver, left and right kidneys, and the healthy tissue was defined as the body covered by the CT scan minus the PTV. The treatment plan was designed to deliver a total dose of 50.4 Gy to the PTV in 1.8 Gy daily fractions, 5 days a week.

### Simulation and optimization

For all patients, simulation was performed on CT scan (RT 16 PRO CT Simulator, General Electrics Systems, Cleveland, OH) with 2.5 mm thick slices. Patients were simulated in the supine position. Four-field 3D-CRT and RapidArc optimization was performed using the treatment planning system Eclipse version 8.9 (Helios, Varian, Palo Alto, CA). Calculation was performed with AAA algorithm, and grid of 2.5 mm.

For 3D-CRT, we selected beam orientation in a way to preserve one kidney in beam eye view (4 beams: anterior (0°), posterior (180°), right (270°) and left (90°). We modified right and left beams in order to avoid totally one kidney at least. Treatment plans were generated using 18 MV photon energy. An 8-mm margin was added to PTV for MLC. The maximum dose rate of 200 MU per minute was selected.

For RapidArc plans, progressive resolution algorithm PRO 8.9.08 was used with 6 MV energy. We used 18 MV for 3D-RT plans to reduce peripheral dose deposit for this deep PTV. With RA, intrinsic value of energy beam is less important due to the number of beams used. The maximum dose rate of 400 MU per minute was selected. Two 360° coplanar arcs sharing the same isocenter and optimized simultaneously were used. These two arcs were delivered with opposite rotation (clock and counter-clock) and so minimize the off-treatment time between the two beams to about 25 seconds. The field size for both arcs was determined by the automatic tool from Eclipse to encompass the PTV, rotation of the collimator was 45° for the first arc, and 315° for the second arc. Collimator was always rotated to a value different from zero in order to avoid tongue and groove effect. Plans were normalized to cover at least 95% of the PTV with ≥ 95% of the prescribed dose. No more than 2% of the PTV was allowed to receive more than 107% of the prescribed dose. Vx is used throughout the analysis to represent the volume receiving "x" Gy or greater dose.

### Statistical methodology and main objective

Dose-volume histograms (DVH) for the target volume and the OAR (right and left kidneys, bowel tract, liver and healthy tissue) were analyzed. The mean and maximum (defined as the maximum dose given to 1% of volume in order to avoid single voxel dose overestimates) doses to the PTV were measured. The volume receiving more than 95% of the prescribed dose (V95), and the average doses to OAR (small bowel, homolateral, contralateral and both kidneys, spinal cord, liver) were also recorded. A non-parametric Wilcoxon matched pair test was used for comparison between the values of 3D-CRT and RA for OAR and PTV. A two-tailed *p *value < 0.05 was used to indicate statistical significance.

Our study aimed to determine whether the 3D-CRT and RapidArc plans allow the treatment to be delivered at the prescribed dose for PTV, without requiring PTV volume adjustment or dose reduction. The main goal was to evaluate the efficiency of both techniques to obtain the lowest dose to the OAR with respect to ideal dose constraints, namely for each kidney:

V10 = 0 Gy, Dmean < 5 Gy, for the spinal cord a maximum dose of 40 Gy, and minimized doses for liver and bowel. Our research that is reported in the manuscript has been performed with the approval of our local ethical committee but was carried out only on dosimetric application and not on humans.

## Results

### PTV

**Figure 1:**
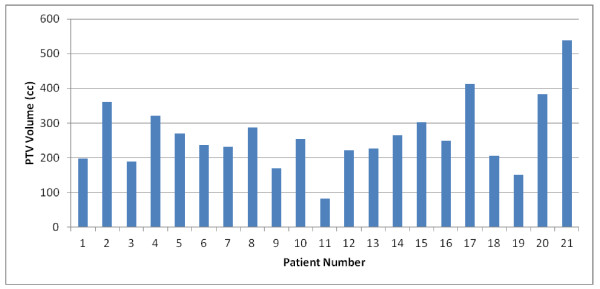
**PTV volume**.

**Figure 2:**
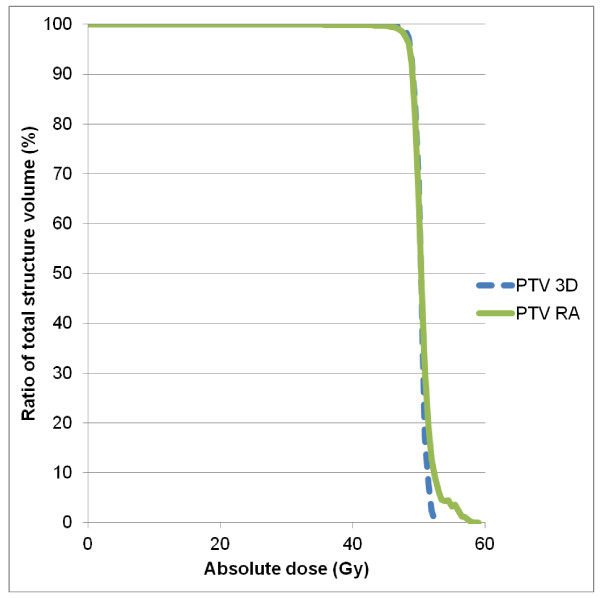
**Average dose-volume histograms (DVH) for the right and left kidney, achieved with RA (solid lines) and 3D-CRT (dashed lines)**.

The mean PTV was 265 ± 100 cm^3 ^(range: 82.4-537.7 cm^3^). Figure 1 shows PTV volumes for all the patients. RA and 3D-CRT plans had excellent coverage of the PTV with at least 95% of the PTV receiving ≥ 95% of the prescribed dose. The DVH for PTV are shown in Figure 2 and results are reported in table 1. Values for the homogeneity index (HI), defined as the (D2-D98)/mean dose ratio, were 0.048 and 0.081 for 3D-CRT and RA, respectively.

**Table 1:** Comparison of PTV between RapidArc (RA) and conventional radiation therapy (3D-CRT) plans

	3D-CRT			RA			
PTV	mean	SD	Range	mean	SD	Range	p-value
Mean dose (Gy)	50.2	0.6	49.2-53.3	50.5	0.9	48.7-51.1	0.5621
D95 (Gy)	48.9	0.8	47.0-49.2	48.6	0.5	46.0-50.0	0.0159
D98 (Gy)	48.1	2.4	38.0-49.4	47.4	2.1	38.6-48.5	0.0007
D2 (Gy)	51.2	0.7	49.5-52.3	52.3	1.4	50.1-57.0	0.0071

### Organs at risk

**Figure 3:**
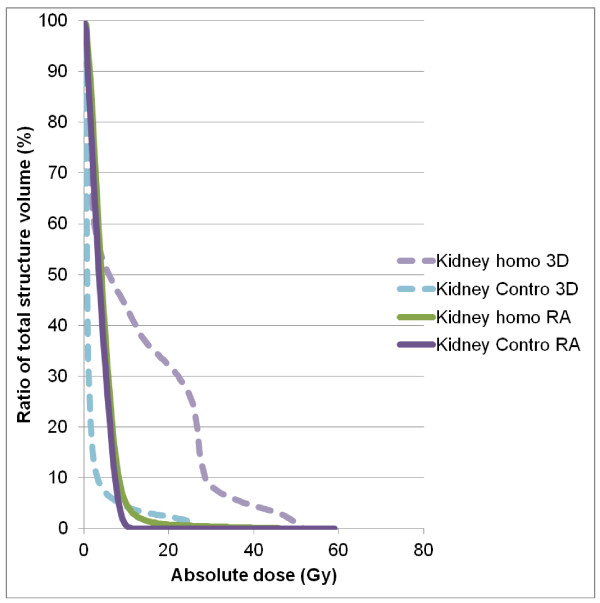
**Average dose-volume histograms (DVH) for the bowel, achieved with RA (solid lines) and 3D-CRT (dashed lines)**.

**Figure 4:**
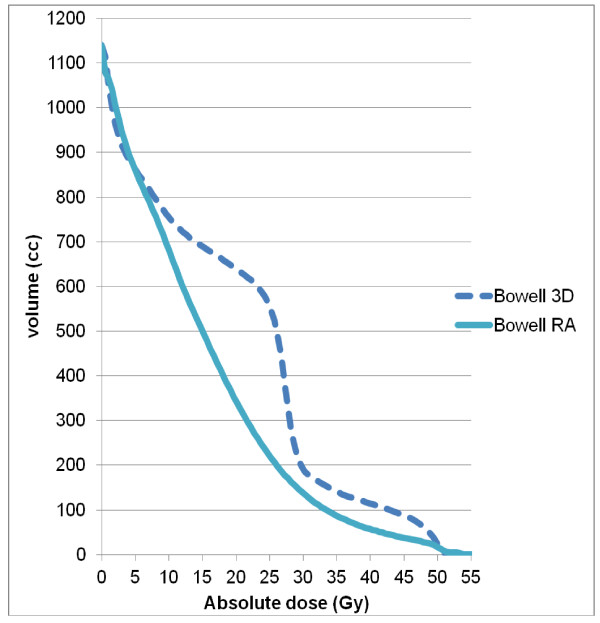
**Average dose-volume histograms (DVH) for the liver and spinal cord, achieved with RA (solid lines) and 3D-CRT (dashed lines)**.

**Figure 5:**
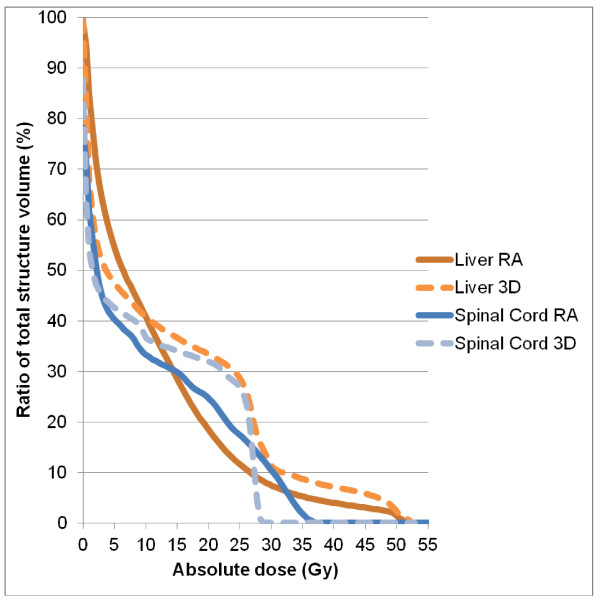
**Average dose-volume histograms (DVH) for the healthy tissue (body -PTV), achieved with RA (solid lines) and 3D-CRT (dashed lines)**.

**Figure 6:**
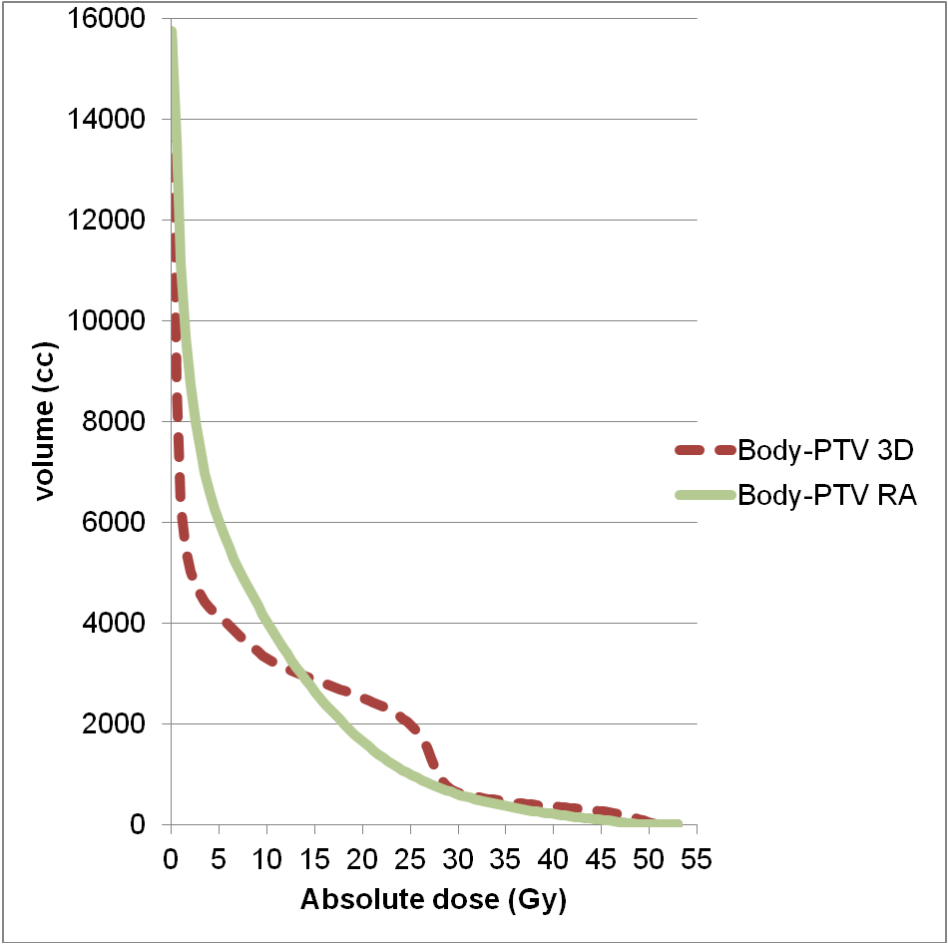
**Average dose-volume histograms (DVH) for the healthy tissue (body -PTV), achieved with RA (solid lines) and 3D-CRT (dashed lines)**.

Table 2 details numerical findings for the kidneys, and table 3 reports the results for the other OAR. RA significantly decreased mean dose (4.7 ± 1.9 vs. 12.6 ± 8.3 Gy), V20 (0.8 ± 2.9 vs. 31.8 ± 24%), V10 (6.4 ± 11.8 vs. 43.6 ± 25.8%), and V5 (40.0 ± 21.5 vs. 52.2 ± 27%) for the homolateral kidney. Conversely, we observed lower mean doses (1.8 ± 2.1 Gy vs. 3.9 ± 0.9 Gy) and V5 values (6.7 ± 11.4 vs. 34.0 ± 18.5%, p < 0.05) for the contralateral kidney with 3D-CRT (Figure 3). The mean dose, V30 and V40 values for bowel were significantly lower with RA compared with 3D-CRT (mean dose: 16.7 ± 6.2 vs. 20.8 ± 6.3 Gy, p = 0.0001, V30 (cc): 135.4 ± 91.3 vs. 180.6 ± 116.6, p = 0.023, V40 (cc): 39.0 ± 42.4 vs. 79.0 ± 72.5, p < 0.0001) (Figure 4). The mean dose (Gy) (10.5 ± 5.4 vs. 12.5 ± 6.3, p < 0.0001) and V30 (%) (8.2 ± 8.5 vs. 11.7 ± 10.8, p: 0.0019) values for liver were significantly lower with RA as compared with 3D-CRT, respectively. The maximum dose allowed to spinal cord was 40 Gy, and this constraint was achieved in 100% of the plans, regardless of the technique (25.5 ± 5.4 for 3D-CRT and 29.6 ± 8.7 for RA) (Figure 5). Dose-volume histograms for healthy tissue (body-PTV) are shown in Figure 6.

**Table 2:** Comparison of renal doses between RapidArc (RA) and conventional radiation therapy (3D-CRT) plans.

	3D-CRT			RA			
	**mean**	**SD**	**Range**	**mean**	**SD**	**Range**	**p-value**

**Homolateral kidney**							
Mean dose (Gy)	12.6	8.3	1.1-31.5	4.7	1.9	2.9-10.2	< 0.0001
V5 (%)	52.2	27.0	4.1-95.9	40.0	21.5	4.3-93.0	0.0033
V10 (%)	43.6	25.8	1.89-90.1	6.4	11.8	0-37.0	< 0.0001
V20 (%)	31.8	24.0	0.0-80.7	0.8	2.9	0.0-13.7	< 0.0001
							
**Controlateral kidney**							
Mean dose (Gy)	1.8	2.1	0.2-8.7	3.9	0.9	1.8-5.6	0.0007
V5 (%)	6.7	11.4	0.0-41.38	34.0	18.5	3.5-80.5	< 0.0001
V10 (%)	3.9	8.1	0.0-30.92	2.2	8.1	0.0-37.3	0.4131
V20 (%)	2.1	4.9	0.0-19.9	0.0	0.0	0.0-0.0	0.1641
							
**Both kidneys**							
Mean dose (Gy)	7.1	4.2	0.9-16	4.3	1.1	2.4-6.9	0.0014

**Table 3:** Comparison of doses to organs at risk between RapidArc (RA) and conventional radiation therapy (3D-CRT) plans

	3D-CRT			RapidArc			
	mean	SD	Range	mean	SD	Range	p-value
**Bowell**							
Mean dose (Gy)	20.8	6.3	10.7-36.4	16.7	6.2	6.5-35.8	0.0001
V30 (cc)	180.6	116.6	12.8-486.2	135.4	91.3	0.0-308.6	0.023
V40 (cc)	79.0	72.5	0.5-201.2	39	42.4	0.0-148.7	< 0.0001
**Liver**							
Mean dose (Gy)	12.5	6.3	2.8-25.0	10.5	5.4	2.3-23.1	< 0.0001
V30 (%)	11.7	10.8	0.0-35.1	8.2	8.5	0.0-28.7	0.0019
**Spinal cord**							
Dmax (Gy)	25.6	5.4	10.0-28.8	29.6	8.7	9.3-37.2	0.0121

**Figure 7:**
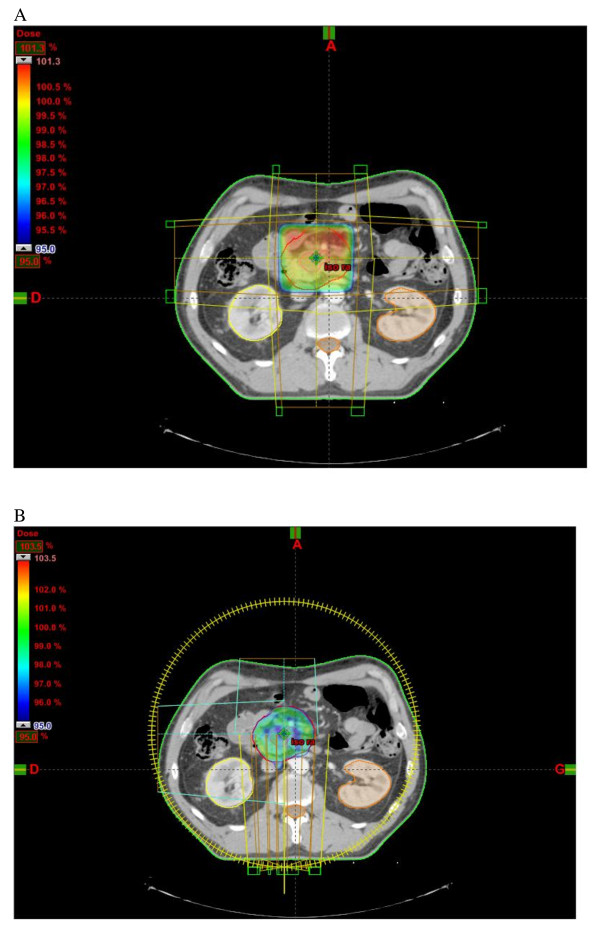
**Poverage of PTV by 95% isodose for A: 3D-RT and B: Rapidarc**.

Representative dose distributions for both techniques are shown in Figure 7.

### Monitor unit and delivery time

The mean number of MU was significantly lower for 3D-CRT: 208.3 ± 13.5 (min-max: 187-241) vs. 569.6 ± 92.8 (min-max: 367-716) (p < 0.0001), and delivery treatment times (defined as the start to the end of the irradiation) were 2 and 2.66 minutes, respectively (NS).

## Discussion

The treatment of pancreatic cancer remains a major clinical challenge. Only 20% of patients are considered eligible for curative surgical resection, whereas radiotherapy given as definitive treatment or adjuvant treatment is a subject of controversy, essentially because of radiation-induced toxicity. It is now generally accepted that RA technology offers the potential to deliver better conformal radiation dose with less toxicity as compared with 3D-RT [9-15]. In the present study, we chose to directly compare 3D-CRT with RA without testing IMRT for two main reasons. Firstly, several studies have already demonstrated benefit of IMRT over 3D-CRT [13-15], and advantage of VMAT over IMRT in terms of conformity and OAR dose reduction [21]. Secondly, in our institution, the RA technique has been implemented as a standard of care for many tumor sites, and is preferred to IMRT because of shorter delivery time. That choice has direct impact on daily practice by improving patient comfort and reducing intrafraction changes in patient position [20].

In this dosimetric analysis, particular emphasis was placed on improving kidney sparing according to the Quantec constraints [22]. Much has changed since the papers by Emami et al., and later Cassady et al., suggesting that total doses of 18-23 Gy and 28 Gy in 0.5-1.25 Gy/fraction may be associated with a 5% and 50% risk of injury at 5 years, respectively [23,24]. Nowadays, conventional treatments are delivered in 2 Gy daily fractions, so we need to take caution with these first published cut-offs. According to current recommendations for partial bilateral kidney radiotherapy, the mean dose should be lower than 15-18 Gy and the V20 lower than 32%, but substantial uncertainty still remains because few published data are available. Moreover, study follow-up periods are usually too short to draw any clear conclusions [22]. RT-induced kidney injury is generally subclinical. Signs and symptoms including arterial hypertension and renal failure usually do not develop until late and their incidence is often underestimated because of a long latency period and the short-life expectancy of the majority of the patients suffering from pancreas cancer. Conventional 3D-CRT allows total preservation of contralateral kidney, but has a detrimental effect on homolateral kidney. In the new era of arctherapy, it is crucial to minimize as much as possible the dose to both kidneys for bilateral preservation. For this reason, we decided to set strong dose constraints for each kidney, i.e. a V10 of 0 Gy and a mean dose < 5 Gy with a successful achievement. In the comparative planning study of VMAT and IMRT conducted by Eppinga et al. in a series of 10 patients with locally advanced pancreatic cancer, doses to the left kidney were significantly reduced with VMAT (V15 of 7.2% and V20 of 2.4%), whereas right kidney sparing was similar with both techniques (V15 of 20.4% and V20 of 9.9% with VMAT). The mean doses delivered to the left and right kidneys were 8.8 and 10.9 Gy, respectively [21].

Our PTV's volumes were smaller. This difference may explain for a part the improvement of OAR sparing. In our present study, the delineation of CTV/PTV corresponds to the new guidelines (manuscript accepted for publication in the Int J Radiat Oncol Biol Phys).

It is unclear if any of these dose and volume parameters could be sufficient to preserve renal function, and the lack of clinical outcome data makes challenging the interpretation of any results. This is the reason why the kidney function of patients requiring abdominal radiotherapy and treated by RA in our institution is now routinely evaluated at baseline. A dimercaptosuccinic acid (DMSA) scintigraphy is performed before and at the end of treatment, and blood tests are carried out during the follow-up period. Since early changes in renal flow are correlated with an increased toxicity, this management offers the possibility to accurately evaluate radiation-associated kidney injury.

Our study demonstrated that kidney sparing can be achieved without compromising the bowel function. Improvements in decreasing dose to the bowel are therefore essential because GI toxicity is the most common acute side effect. Devisetty et al. showed a significant higher acute GI toxicity for V30 > 450 cm^3 ^than for V30 ≤ 450 cm^3 ^(33% vs. 8%, p = 0.003, respectively) [25]. Recommendations for V45 < 195 cm^3 ^when peritoneal cavity was delineated were reported by Kavanagh et al. [26,27]. We delineated the bowel as the peritoneal cavity, and obtained a V30 < 200 cm^3 ^and a V40 < 55 cm^3 ^with RA, which is very promising for reducing acute digestive toxicity. Dosimetric data with respect to the liver complied with the Quantec recommendations (i.e. mean dose < 30-32 Gy) for both techniques with reported mean doses of 11.0 Gy and 9.7 Gy with 3D-CRT and RA, respectively [27].

Despite these positive results, the IMRT/RA techniques raise concerns about the increased number of MU required, and the low-dose radiation delivered to healthy tissue compared with 3D-RT treatment leading to a potential increase of the risk of secondary malignancies [28,29]. In this study, we noted superiority of 3D-CRT over RA for doses inferior to 15 Gy, but for "middle doses" (> 15Gy) healthy tissue doses were decreased by RA. The real impact and the dose level potentially incriminated in the risk of developing a second cancer are still unclear.

## Conclusion

The RA technique results in better OAR sparing than 3D-CRT for pancreatic and bile duct malignancies, especially for small bowel and kidney preservation, while covering target volume, making the rationale for use of radiotherapy in these diseases more solid and less controversial. These improvements have led us to implement rapidly this technique into the clinic as a standard of care for pancreatic and bile duct cancers and after 4 to 6 cycles of chemotherapy.

## Conflict of interest notification

The authors declare that they have no competing interests.

## Authors' contributions

SV, PF conceived the study, collected data, and drafted the manuscript. NA, JBD, OR and DA participated in coordination and helped to draft the manuscript. DA provided mentorship and edited the manuscript. All authors have read and approved the final manuscript.
